# Health recommender systems to facilitate collaborative decision-making in chronic disease management: A scoping review

**DOI:** 10.1177/20552076241309386

**Published:** 2025-01-06

**Authors:** Antonia Barbaric, Kenneth Christofferson, Susanne M Benseler, Chitra Lalloo, Alex Mariakakis, Quynh Pham, Joost F Swart, Rae S M Yeung, Joseph A Cafazzo

**Affiliations:** 1 Institute of Health Policy, Management, and Evaluation, 7938 University of Toronto, Toronto, ON, Canada; 2 Centre for Digital Therapeutics, 7989 University Health Network, Toronto, ON, Canada; 3 Department of Computer Science, 7938 University of Toronto, Toronto, ON, Canada; 4 Department of Pediatrics, 70401 Cumming School of Medicine, University of Calgary, Calgary, AB, Canada; 5 Children's Health Ireland, Dublin, Ireland; 6 Child Health Evaluative Sciences, 7979 The Hospital for Sick Children, Toronto, ON, Canada; 7 Telfer School of Management, 6363 University of Ottawa, Ottawa, ON, Canada; 8 Department of Pediatric Rheumatology and Immunology, Wilhelmina, Children’s Hospital, University Medical Center Utrecht, Utrecht, The Netherlands; 9 Faculty of Medicine, 8125 Utrecht University, Utrecht, The Netherlands; 10 Department of Immunology and Medical Science, 7938 University of Toronto, Toronto, ON, Canada; 11 Division of Rheumatology, 7979 The Hospital for Sick Children, Toronto, ON, Canada

**Keywords:** Health recommender system, chronic disease, decision-making, collaborative, behavior change lifestyle, digital therapeutics

## Abstract

**Objective:**

Health recommender systems (HRSs) are increasingly used to complement existing clinical decision-making processes, but their use for chronic diseases remains underexplored. Recognizing the importance of collaborative decision making (CDM) and patient engagement in chronic disease treatment, this review explored how HRSs support patients in managing their illness.

**Methods:**

A scoping review was conducted using the framework proposed by Arksey and O’Malley, advanced by Levac et al., in line with the PRISMA-ScR checklist. Quantitative (descriptive numerical summary) and qualitative (inductive content analysis) methods wered used to synthesize the data.

**Results:**

Forty-five articles were included in the final review, most commonly covering diabetes (9/45, 20%), mental health (9/45, 20.0%), and tobacco dependence (7/45, 15.6%). Behavior change theories (10/45, 22.2%) and authoritative sources (10/45, 22.2%) were the most commonly referenced sources for design and development work. From the thematic analysis, we conclude: (a) the main goal of HRSs is to induce behavior change, but limited research investigates their effectiveness in achieving this aim; (b) studies acknowledge that theories, models, frameworks, and/or guidelines help design HRSs to elicit specific behavior change, but they do not implement them; (c) connections between CDM and HRS purpose should be more explicit; and (d) HRSs can often offer other self-management services, such as progress tracking and chatbots.

**Conclusions:**

We recommend a greater emphasis on evaluation outcomes beyond algorithmic performance to determine HRS effectiveness and the creation of an evidence-driven, methodological approach to creating HRSs to optimize their use in enhancing patient care.

**Lay summary:**

Our work aims to provide a summary of the current landscape of health recommender system (HRS) use for chronic disease management. HRSs are digital tools designed to help people manage their health by providing personalized recommendations based on their health history, behaviors, and preferences, enabling them to make more informed health decisions. Given the increased use of these tools for personalized care, and especially with advancements in generative artificial intelligence, understanding the current methods and evaluation processes used is integral to optimizing their effectiveness. Our findings show that HRSs are most used for diabetes, mental health, and tobacco dependence, but only a small percentage of publications directly reference and/or use relevant frameworks to help guide their design and evaluation processes. Furthermore, the goal for most of these HRSs is to induce behavior change, but there is limited research investigating how effective they are in accomplishing this. Given these findings, we recommend that evaluations shift their focus from algorithms to more holistic approaches and to be more intentional about the processes used when designing the tool to support an evidence-driven approach and ultimately create more effective and useful HRSs for chronic disease management.

## Introduction

### Background

Accurate and timely treatment decisions are essential to everyday clinical practice. In evidence-based medicine, decisions are often made by choosing from a set of options based on clinician expertise and supportive test results, patient preferences, and established scientific knowledge.^1^ Depending on the context and type of decision, there are a variety of ways in which patients can participate in the decision-making process; models of patient engagement in decision-making include a continuum of patient involvement ranging from paternalistic (physician directed with passive patient) to collaborative (support from physician with proactive patient).^2^ In the context of chronic diseases, a collaborative approach is preferred since decisions are mostly focused on management of symptoms to improve or maintain health conditions, all of which require patient engagement.^3^

A variety of digital technologies designed to improve clinical decision-making have emerged in recent years, such as clinical decision support systems (CDSSs). This technology is integrated with electronic medical records and is used at the point of care by clinicians. While numerous studies have shown its efficacy in augmenting clinicians in their decision-making processes,^4^ its workflow lacks support for patient engagement. In contrast, health recommender systems (HRSs) represent another category that support decision-making. HRSs are designed to be accessible by both patient and clinician, thus enabling more collaboration between stakeholders. HRSs cover a larger range of health domains that are relevant to patients, such as supporting healthier lifestyles through exercise and dietary plans, domains not supported by CDSSs. HRSs can also have similar functionalities to CDSSs, including recommending therapies and medications for specific illnesses, but are outputted in a way that is accessible to the patient (i.e. does not require vast clinician knowledge).^5^

Recent HRS scoping reviews have primarily focused on their use across all applicable health domains. One review consolidated HRS development, usage, and evaluation evidence from the last decade,^6^ while another review created an evidence map encompassing development and evaluation details.^7^ In both reviews, general HRS attributes were collected (i.e. specific health domain, intended users, and recommendation technique), and as a result, had a lesser focus on the specific details surrounding design and evaluation processes.

Despite the increased use of recommender systems in healthcare and their broad reach, the extent of their use and purpose in patient-facing chronic disease management is less well known, limiting their usefulness as a collaborative tool. Additionally, HRS studies often focus only on algorithmic details and performance; evaluations of the HRS on a systems level are limited. As a result, the practices for designing and evaluating effective HRSs for chronic disease management remain inconsistent and incomplete. To address this gap in knowledge, this review will serve as a first step toward a comprehensive HRS development framework. It will synthesize the evidence base of HRS use in chronic disease management for patients. A HRS development framework would help to establish a structured, methodological approach to design and build HRSs so that they are effective in fostering collaborative decision-making involving both patients and healthcare professionals.

### Research question and aims

The central research question for this scoping review was: What is known in the literature regarding the use of HRSs to help patients with chronic disease manage their illness? The aims were to identify the following attributes: (a) key HRS design characteristics (to be used as a way to determine the level of personalization for users); (b) the main tools and processes used during HRS design and development; and (c) the intended use of the HRS, including the extent of integration into clinical practice.

## Methods

### Review framework

The method framework outlined by Arksey and O’Malley,^8^ advanced by Levac et al.,^9^ was utilized for this scoping review. The following five main steps were followed: (a) identifying the research question; (b) identifying relevant studies; (c) study selection; (d) charting the data; and (e) collating, summarizing, and reporting the results. Levac et al.^9^ provide methodological recommendations for each stage to further enhance and clarify the foundational framework developed by Arksey and O’Malley. This approach was selected since it allowed researchers to showcase a specific research field by summarizing the characteristics of relevant primary research. The research question (step 1) was detailed above, and the subsequent steps are discussed below.

### Step 2: identifying relevant studies

This review was guided by the PRISMA-ScR (Prisma Extension for Scoping Reviews) checklist.^10^ A protocol was followed, but not registered. Evidence synthesis: To identify all relevant primary research publications, a comprehensive list of interdisciplinary databases was searched including Medline, EMBASE, PyschInfo, Scopus, IEEE, and ACM. Strategy: Two reviewers (AB and KC) independently searched all databases, using key terms related to the following concepts: “recommender system,” “decision making,” and “chronic disease.” An iterative approach was taken with the search strategy; first, two to four key terms for each concept were brainstormed and applied to searches performed in Medline and Scopus. Based on the number of results returned and the content from some abstracts (five to seven) that were preliminarily screened, the strategy was then tailored by excluding and/or including relevant key terms. Each reviewer screened reference lists of all relevant systematic and scoping reviews to identify any publications not otherwise captured in our original search. The search for relevant studies took place between June and August 2023 and was updated in July 2024. Due to the relatively recent emergence of HRSs, no time constraints were applied to the search.

### Step 3: study selection

The results retrieved from Step 2 were saved in a reference management system, Covidence, which automatically detected and removed duplicate search results. Given that this is a scoping review, results were selected based on inclusion and exclusion criteria and were not evaluated based on their scientific quality and rigor. Like Step 2, the selection criteria used for this review underwent an iterative process based on a set of studies (approximately 10 to 15) that were screened in the preliminary search. The final inclusion and exclusion criteria are listed below:

Publications were included in the scoping review if they met all of the following inclusion criteria: (a) target HRS population entailed individuals with a specific chronic disease (including mental health and substance use); (b) publication presents a performance evaluation of the HRS or the methods used to design the HRS; (c) HRS provides recommendations that are meant for the individual with chronic disease; (d) data inputs for the HRS are manually inputted from the user and/or are loaded in from databases containing personal health information; (e) the study is published in English; and (f) the full text of the publication is available.

Publications were excluded from the scoping review if they met any of the following exclusion criteria: (a) the publication primarily focuses on describing details of the algorithms or datasets related to the HRS (e.g. optimization); (b) the publication does not identify and/or describe the key design components/features of the HRS; (c) HRS is designed for a health domain that is not a chronic illness; (d) HRS is designed for pharmaceutical companies or clinician purposes that are not patient facing (workflows, information retrieval, lab reports, etc.); (e) HRS is designed to diagnose patients or classify disease(s); and (f) the study is a systematic review, scoping review, gray literature (i.e. material that is not peer reviewed), book chapter, or an abstract.

Two reviewers (AB and KC) independently screened all articles in both rounds. The first round involved reviewing the title and abstract of all articles retrieved in Step 2 and deciding whether to include or exclude each one based on the criteria listed above. If a reviewer was unsure about a specific article, it was advanced to the next screening round. In the second round, the articles were screened again for the inclusion and exclusion criteria, but this time according to their abstract and full text. Reviewers had the opportunity to vote “Maybe” on Covidence if they were not certain whether a specific article should be included. All the articles voted as “Maybe,” as well as any articles with discrepancies in the vote (i.e. disagreements) were discussed between reviewers to reach consensus.

### Step 4: charting data

A data charting form was developed on Covidence and used by two reviewers (AB and KC) independently to extract all relevant data. The preliminary form was organized into two main sections: Study characteristics and HRS design characteristics. Study characteristics identified the following components: year of publication, lead author, location of study, and research methodology. HRS design characteristics captured health domain, intended user, HRS output type, data inputs, source of data inputs, and theories used in the design process. A descriptive-analytical method was used to extract all relevant data from each study.^9^ After testing the form on a set of four to six articles, edits were made to the different types of categories used for the HRS design characteristics, specifically by adding, removing, and redefining categories relevant to the attributes articulated in the HRS design characteristics section (such as health domain, intended user, HRS output type, data inputs, source of data inputs, and theories used in the design process). Any discrepancies between the two reviewers were flagged and discussed until consensus was reached.

### Step 5: summarizing and reporting the results

Results for this study were organized into quantitative and qualitative findings. The quantitative results consist of the study and HRS design characteristics, which were represented using a descriptive numerical summary approach and are presented in the tabular format below. The qualitative findings capture overarching themes from the main findings of the included studies. An inductive, qualitative content analysis was used for the Results and Discussion sections of each publication to identify codes from all the study's results. These codes were then organized into thematic groups and presented in a way that relates to the main research question.

## Results

### Quantitative findings

**Figure 1.**
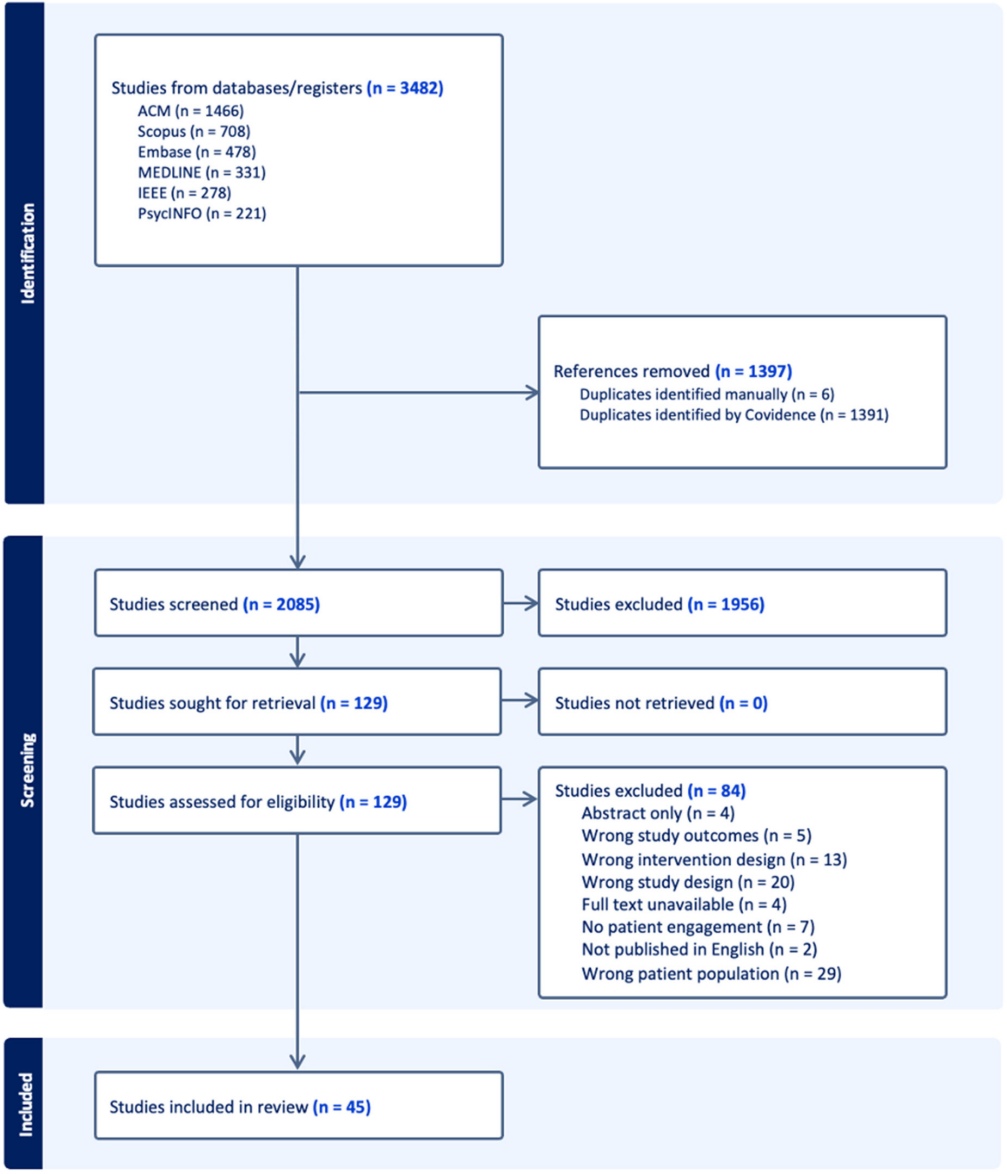
Flow diagram of study selection process *(generated by Covidence software)*.

A total of 3482 articles were retrieved from six databases and imported for screening. 1397 articles were identified as duplicates and removed, and the remaining 2085 articles were screened by two reviewers (AB and KC). Both AB and KC scanned the articles by reviewing titles and abstracts, assessing relevance according to the inclusion and exclusion criteria. After the first pass, 1956 articles were consensually removed, and 120 articles underwent a full-text review by both AB and KC. From the 129 studies, 84 were excluded due to the following reasons: the study population was not patients with chronic illness (29/84, 34.5%; applicable exclusion criteria: 4), the study design did not describe and/or evaluate the HRS (20/84, 23.8%; applicable exclusion criteria: 1 and 2), the HRS was not related to management of chronic illness (13/84, 15.5%; applicable exclusion criteria: 3 and 5), no patient involvement (7/84, 8.3%; applicable exclusion criteria: 1 and 4), the study outcomes did not match the scoping review criteria (5/84, 6.0%; applicable exclusion criteria: 1 and 2), the full text was unavailable (4/84, 4.8%; study limiter), only abstracts of the research were published (4/84, 4.8%; applicable exclusion criteria: 6), and the publication was not written in English (2/84, 2.4%; study limiter). In the end, 45 articles were included in the scoping review (Figure 1). The quantitative findings outlined in this section address the first two aims of this study (i.e. key HRS design characteristics, and the main tools and processes used during HRS design and development) and are captured in Tables 1–3.

**Table 1.** Frequency of publication (*N* = 45).

Year of publication	Studies, *n* (%)	References
2010	1 (2.2)	^11^
2013	2 (4.4)	^12,13^
2015	1 (2.2)	^14^
2016	2 (4.4)	^15,16^
2017	5 (11.1)	^17–21^
2018	6 (13.3)	^22–27^
2019	6 (13.3)	^28–33^
2020	5 (11.1)	^34–38^
2021	6 (13.3)	^39–44^
2022	6 (13.3)	^45–50^
2023	3 (6.7)	^51,52,53^
2024	2 (4.4)	^54,55^

**Table 2.** Study characteristics (*N* = 45).

Study characteristics	Studies, *n* (%)	References
Study location		
United States	16 (35.6)	^13,15,16,22,29,30,35,36,40,42,45–47,49,51,52^
Germany	3 (6.7)	^20,41,50^
Spain	3 (6.7)	^11,28,33^
China	4 (8.9)	^19,27, 53,55^
Ecuador	2 (4.4)	^18,24^
India	2 (4.4)	^32,43^
Portugal	2 (4.4)	^34,37^
Taiwan	2 (4.4)	^14,31^
Canada	1 (2.2)	^26^
Colombia	1 (2.2)	^48^
Denmark	1 (2.2)	^39^
Greece	1 (2.2)	^23^
Ireland	1 (2.2)	^12^
New Zealand	1 (2.2)	^38^
Pakistan	1 (2.2)	^21^
Russia	1 (2.2)	^25^
Scotland	1 (2.2)	^56^
United Kingdom	2 (4.4)	^44,54^
Research methodology		
Design papers (some containing preliminary, brief evaluations)	20 (44.4)	^11,13,18–20,25,26,30–33,36–38,41,43–45,50,56^
Pilot study	6 (13.3)	^14,23,28,34,35,48^
Observational (includes both retrospective and/or longitudinal)	5 (11.1)	^22,40,42,46,52^
Randomized controlled trial (RCT)	3 (6.7)	^15,16,51^
Usability study	3 (6.7)	^24,29,53^
Feasibility	3 (6.7)	^21,39,54^
User-centered design process (containing: interviews, low-fidelity prototypes, and usability evaluation)	1 (2.2)	^12^
Case vignettes	1 (2.2)	^27^
Proof of concept	2 (4.4)	^47,55^
“Recommendation” paper (principles of HRS)	1 (2.2)	^49^
Type of user involvement		
Active involvement: participates in clinical trial	17 (37.8)	^12,14–16,22–24,28,29,34,35,39,48,51,53,55,56^
Passive involvement: used for data purposes only	12 (26.7)	^19–21,27,31,32,42,43,46,47,52,53^
No user involvement: not applicable given the purpose of a publication (e.g. design paper)	10 (22.2)	^11,25,30,36–38,41,45,49,50^
Active involvement: provides feedback via HRS (ratings) or surveys	5 (11.1)	^13,16,18,40,54^
Simulated data/virtual subjects	3 (6.7)	^26,33,44^

**Table 3.** Health recommender system design characteristics.

Design characteristics (*N* = 45)	Studies, *n* (%)	References
Health domain		
Diabetes	9 (20.0)	^11,18,19,32,33,37,43,46,56^
Mental health	9 (20.0)	^14,22,27,36,39,41,47,52,54^
Tobacco dependence	7 (15.6)	^13,16,30,31,35,40,51^
Physical or motor disability (requiring long-term rehabilitation)	4 (8.9)	^23,28,44,49^
Cancer	3 (6.7)	^23,29,45^
Cardiac health	2 (4.4)	^21,25^
Psoriasis	2 (4.4)	^20,50^
Obesity	2 (4.4)	^15,34^
Obstructive sleep apnea	1 (2.2)	^42^
Dementia	2 (4.4)	^12,53^
Eating disorder	1 (2.2)	^48^
Asthma	1 (2.2)	^24^
Kidney/end stage renal disease	1 (2.2)	^55^
General purpose	1 (2.2)	^38^
Intended user		
Patient	28 (62.2)	^11,14–16,22,23,25,27,29–33,35,37–43,46,47,51,52,54–56^
Patient with clinician	12 (26.7)	^12,13,18–21,24,28,44,48–50^
Patient with caregiver	4 (8.9)	^26,34,45,53^
Patient with caregiver and clinician	1 (2.2)	^36^
HRS output type		
Lifestyle (e.g. diet and physical activity)	12 (26.7)	^11,18,26,28,32,37,43,44,48,49,55,56^
Motivational messaging	9 (20.0)	^13,15,16,25,30,31,35,40,51^
Interventions specific to chronic disease	10 (22.2)	^14,21,27,36,39,41,46,47,53,54^
Educational	7 (15.6)	^12,23,24,29,34,45,52^
Drug intervention	6 (13.3)	^19,20,33,38,42,50^
Digital health intervention	1 (2.2)	^22^
Data inputs		
Health profile (physical health, medical tests, medical history)	27 (60.0)	^11,15,18–21,23–27,29,31–33,36,41–46,48,50,52,56^
User demographic (e.g. age, gender, race, location of residence, and occupation)	18 (40.0)	^12,14,16,18–21,26,28,31,32,37,39,46,48,50,53,56^
Lifestyle (e.g. diet, physical activity, and health goals)	18 (40.0)	^11,12,16,18,23,25–27,33,34,37,41,46,53,55,56^
Feedback about recommendation (e.g. ratings and app usage time)	13 (28.9)	^12–14,16,23,28,30,35,37,40,47,51^
Mental health metrics (e.g. standardized questionnaires)	5 (11.1)	^25,27,36,39,52^
Unspecified	2 (4.4)	^15,22,49^
External Datasource	1 (2.2)	^38^
Source of data inputs		
User (can be defined as: patient, clinician, caregiver)	33 (73.3)	^12–16,18,24–30,32,34–40,43–48,52–56^
Datasets of past user activity	11 (24.4)	^16,18,24,30,35,38,40,45,47,51,56^
Device: wearables, medical devices	6 (13.3)	^14,25,28,33,42,44^
Unknown	6 (13.3)	^11,22,23,31,41,49^
Electronic health records	4 (8.9)	^19–21,50^
External knowledge base	1 (2.2)	^55^
Theories guiding HRS design		
Behavior change theory	10 (22.2)	^15,16,25,30,31,34,35,39,41,51^
Guidelines from authoritative sources	10 (22.2)	^16,18,27,30,35,36,40,50,51,53^
None	26 (57.8)	^11,13,14,17,20–24,26,29,32,33,37,38,42–49,52,54,55^
User-centered design approach	1 (2.2)	^12^
International Patient Decision Aid standard	1 (2.2)	^19^
Three-step shared decision-making model	1 (2.0)	^19^
Gamification	1 (2.0)	^28^

## Study characteristics

### Publication frequency

The first study relevant to this scoping review emerged in 2010, and since 2015 there has consistently been at least one article published every year. A rise in the number of publications began in 2017, with five articles published (5/45, 11.1%). Since then, six articles have been the highest number published within a year, which occurred in 2018, 2019, 2021, and 2022 (6/45, 13.3% per year).

### Study location

Many of the included studies were performed in the United States (16/45, 35.6%); other countries with more than one study included China (4/45, 8.9%), Germany (3/45, 6.7%), Spain (3/45, 6.7%), Ecuador (2/45, 4.4%), India (2/45, 4.4%), Portugal (2/45, 4.4%), Taiwan (2/45, 4.4%), and United Kingdom (2/45, 4.4%).

### Research methodology

A variety of research methods were used among the publications included in the scoping review. Many of the publications (20/45, 44.4%) did not report using a specific research methodology and were therefore classified as “design papers” since they typically presented details about an HRS and/or performed very preliminary evaluations. Similarly, one publication published by Ran et al.^49^ was a recommendation paper, meant to share key principles of HRS design more generally. Other common types of methodology included pilot studies (6/45, 13.3%), observational cohort studies (retrospective or longitudinal) (5/45, 11.1%), randomized controlled trials (RCTs) (3/45, 6.7%), usability studies (2/45, 4.4%), and feasibility studies (2/45, 4.4%). Methods used in only one paper include user-centered design (1/45, 2.2%), case vignettes (1/45, 2.2%), hybrid-effectiveness (1/45, 2.2%), and proof of concept (1/45, 2.2%). Echoing the growing prevalence of publications in this space, most of the methodologies still consist of preliminary, formative evaluations and are not yet at the level of maturity to perform an RCT.

### Type of user involvement

The extent of user involvement in each study was also captured and categorized into one of five main categories, ranging on a continuum of passive to active involvement: no user involvement in design or evaluation phases, use of only simulated data and/or virtual subject, use of real user data for back-end algorithmic functionality, real user participation via feedback from HRS or survey, and real user participation via clinical trial.

Two of the categories are described as active user involvement: either the participant was enrolled in a clinical trial over a period of time (17/45, 37.8%), or they only participated by providing feedback through HRS output ratings or questionnaires (5/45, 11.1%). The first category requires the user to be more involved in the research process since they are asked to provide feedback about the HRS design, whereas the second category collects only limited information from the user. Three groups of authors—Sadasviam et al., Hors-Fraile et al., and Ramesh et al.—had studied with both active participants and feedback collection from HRS output ratings, so they were counted twice. Participants were considered to be passively involved if they only relied on existing data in the study rather than generating more data from feedback or other active methods (12/45, 26.7%). The fourth category involved no real user involvement and instead consisted of using simulated data or virtual subjects (3/45, 6.7%); Yang et al.^27^ used both passive data and simulated data in their publication and was therefore counted twice. The last category did not require user involvement given the nature and purpose of the publication, such as a design paper (10/45, 22.2%). Many of the publications involved either active or passive user involvement; most of the observational studies involved passive user involvement while the other methods, such as usability and pilot studies, required active user involvement. Lastly, not all publications that described active user involvement presented the subsequent data in the publication but were still classified as active participation.

### HRS design characteristics

#### Health domain

Given that this scoping review is focused on chronic illness, only those health domains that are explicitly related to a chronic disease were included. The three most common health categories were diabetes (9/45, 20.0%), mental health (9/45, 20.0%), and tobacco dependence (7/45, 15.6%). For diabetes, two out of the nine articles (2/9, 22.2%) focusing on diabetes were general (unspecified type), another two focused (2/9, 22.2%) on type 1, and the rest (5/9, 55.5%) focused on type 2. The mental health publications can be further categorized into the following: general mental health (5/45, 11.1%), depression (2/45, 4.4%), emotional disorder (1/45, 2.2%), and anxiety (1/45, 2.2%). The rest of the articles included in this review had diverse areas of focus. There were four publications pertaining to physical and/or motor disability that require long-term care; one out of the four focused (1/4, 25%) on assistive device technology, while the other three (3/4, 75%) were focused on rehabilitation exercises (two of which were for cardiac patients). Three articles (3/45, 7.1%) presented recommender systems (RSs) for cancer patients; one article covered multiple forms of cancer, another article was specifically related to breast cancer, and the third article focused on ovarian cancer for females. The rest of the health domains included cardiac health (2/45, 6.7%), psoriasis (2/45, 6.7%), obesity (2/45, 6.7%), obstructive sleep apnea (1/45, 2.2%), dementia (1/45, 2.2%), eating disorders (1/45, 2.2%), asthma (1/45, 2.2%), kidney/end-stage renal disease, and general-purpose chronic illness, which included areas related to ADHD and abdominal distension (1/45, 2.2%).

#### Intended user

In contrast to the type of user involvement (as described in the Study characteristics section), this characteristic is meant to describe the population for whom the HRS is designed for. While this review is scoped around patient-facing HRSs, there are slight variations that exist within this space according to the following categories: patients (28/45, 62.2%), patients with caregivers (4/45, 8.9%), patients with clinicians (12/45, 26.7%), and patients with both their caregivers and clinicians (1/45, 2.2%). HRSs that involved users in addition to patients either created different interfaces for each stakeholder group or included additional support for non-expert users.

#### HRS output type

Based on the HRS descriptions, the output for each can be categorized into one of the main six types: lifestyle, motivational messaging, chronic disease-specific therapy, educational, drug intervention, and digital health intervention. Lifestyle is defined as a user's behavior as it relates to their diet and physical exercise and was the output for 12 of the 45 articles (12/45, 26.7%). Motivational messaging involves elements that can be described as encouraging, instructional, motivational, and/or cautionary; these types of HRS outputs were described in nine articles (9/45, 20.0%). Chronic disease-specific therapy includes any treatments that are prescribed for specific illnesses only and were found in 10 articles (10/45, 22.2%). Educational recommendations can be described as providing relevant knowledge to the user and were the output for seven articles (7/45, 15.6%). Pharmaceutical drugs were the output of HRSs that recommended drug interventions, which comprised 6 out of the 45 articles (6/45, 13.3%). Only 1 out of the 45 (1/45, 2.2%) articles exclusively offered digital health interventions (DHI)^57^ as the HRS output.

#### Data inputs

Across the HRSs included in this review, a variety of data types are used as part of the system inputs. At a minimum, many publications included data related to patient demographic with the main metrics being age, gender, and race (18/45, 40.0%). Other categories involve data types that describe a patient's health profile (27/45, 60.0%), such as weight, blood pressure, body temperature, as well as past medical history and any relevant medical tests. Beyond a patient's physical health profile, inputs relating to mental health were also captured (5/45, 11.1%) and included data such as depression, anxiety, and mood levels. Information about a patient's lifestyle (behaviors and actions) was also collected from 18 articles, mostly pertaining to physical activity and dietary habits, personal interests, and preferences and goals (18/45, 40.0%). Metrics from the HRS pertaining to user feedback, such as ratings related to the recommendation, and time spent on the app also served as data inputs, as outlined in 13 articles (13/45, 28.9%). Lastly, one article used information from an external dataset which served as the source of their data inputs (1/45, 2.2%), and two articles did not specify where the data inputs were coming from (2/45, 4.4%). It is important to acknowledge that this categorization reflects only the type of data inputs used by each HRS and does not specify the quantity of data collected in each category type. Furthermore, these categorizations are based solely on explicit information shared in each publication, so any additional data collected by the authors that are not mentioned may not be represented in this summary.

#### Source of data inputs

Complementary to the types of data inputs are the sources from which they are retrieved. Similar to the diverse set of data inputs, seven main categories can describe the source of these data: user, databases containing past user data, physician inputs, devices, health records, external databases, and unspecified. User input involves the patient and/or clinician manually inputting the data when using the HRS (33/45, 73.3%). Other sources of data collection include databases containing previous user activity (11/45, 24.4%). Data retrieved from devices can include medical devices (such as the CPAP machine in Araujo et al.^42^) as well as wearables that passively collect user data (6/45, 13.3%). There was also a set (6/45, 13.3%) of articles that did not specify from where the data were retrieved. When considering various databases, four articles specifically used EHR access as a means to retrieve relevant information (4/45, 8.9%). Lastly, only one article (1/45, 2.2%) used an external knowledge base to retrieve relevant data, specifically by using a large language model.^55^

#### Tools guiding the HRS design and development process

Among the studies that reported the use of theories, models, frameworks, and/or guidelines to inform their design and development processes, behavior change theories (BCTs) and guidelines from authoritative sources were the most referenced (10/45, 22.2%) and (10/45, 22.2%), respectively. The health domains of articles referencing BCTs include tobacco dependence (5/10, 50.0%), obesity (2/10, 20.0%), mental health (2/10, 20.0%), and hypertension (1/10, 10.0%). Authoritative sources include domain experts and guidelines, standardized questionnaires, and academic publications; health domains referring to these types of data include tobacco dependence (5/9, 55.5%), mental health (3/10, 30.0%), diabetes (1/9, 11.1%), and psoriasis (1/9, 11.1%). Most of the other articles did not specify whether any tools were used to inform the content development, front-end, or development of the presented HRS (26/45, 57.8%). Tools that were referenced only by one article include the user-centered design process (1/45, 2.2%), International Patient Decision Aid standard (1/45, 2.2%), three-step shared decision-making model by Elwyn et al.^58^ (1/45, 2.2%), and gamification theory (1/45, 2.2%).

## Qualitative findings: thematic analysis

The findings in this section relate to the second and third aims of this study (i.e. main tools used and processes undertaken during HRS design and development and the intended use of the HRS). A thematic analysis is presented which was derived using an inductive, qualitative content analysis approach based on each of the included study's main findings.

### The main goal of HRSs is to induce behavior change, but limited research investigates their effectiveness in achieving this aim

In this scoping review, the top health domains HRSs were designed for included mental health, diabetes, and tobacco dependence, collectively making up over 50% of the selected publications. Regardless of the specific advice given by an HRS, whether it be receiving motivational messaging, adopting an app, engaging with a new hobby, or participating in relevant clinical treatments, it inherently aims to induce a change in the user's behavior. Despite this end goal, the aim for all of these studies was focused on outcomes relating to feasibility or presenting a conceptual design of the HRS. A similar observation can be made for the latter half of publications in this scoping review. One potential explanation for the lack of focus on HRS effectiveness is because the work in this field is still formative and in the early stages of product development. Only one publication, authored by Cheung et al.,^22^ explicitly focused on the HRS’s ability to enact behavior change by exploring the effects it had on the usage of specific mental health apps.

Despite the lack of focus, there is at the very least acknowledgment for the importance of HRS continued use and adoption. The work published by Gmez-Portes et al.^44^ primarily focuses on the technical aspects of the HRS algorithm but concludes by stating that continued use of the HRS tool is important in the context of improving physical rehabilitation therapy at home. Similarly, González-González et al.^28^ investigated the impact of an HRS on user motivation to receive therapy but did not have sufficient results to share any definitive conclusions. Furthermore, there is also research investigating the impact specific patient attributes have on HRS adoption and continued use. Chaturvedi et al.^52^ identified that both age and gender were sensitive to different recommendation methods; in their study, they identified that males over the age of 35 were the most responsive to their recommendation method. Faro et al.^35^ specifically compared HRS use between users of different race (Caucasian versus African American), with the intent of future work personalizing the HRS for specific subpopulations. Lastly, Chen et al.^40^ concluded that HRSs may be more well suited for “harder to reach” and “harder to engage” groups.

Across publications, there is agreement that personalized content creates opportunity for effective HRSs, therefore leading to sustained use of the tool. HRS RCTs, one of the more robust evaluation methodologies in this review, involved systems that provided personalized motivational messaging for tobacco dependence (two publications) and obesity (one publication). Findings from Hales et al.^15^ indicate that the HRS group lost significantly more weight compared to the control group. Similarly, Sadasivam et al.^16^ found a significantly higher proportion of days of user acceptance of messages, compared to the control group. Both Sadasivam et al. and Faro et al.^51^ found no significant differences when it came to actual smoking behavior.

### Studies acknowledge theories, models, frameworks, and/or guidelines to help design HRSs to elicit specific behavior change, but they do not implement them

For an HRS to be considered effective, some type of behavior change should be elicited. Of the publications that mentioned use of theories, models, frameworks, and/or guidelines (23/45, 56.7%), the most referenced were BCTs (10/23, 43.5%). While the majority of publications in this review did not explicitly describe if and how they used BCT, at the very least they recognized the importance of behavior change for increasing personalization and the use of such theories to elicit a specific outcome (such as adherence) when using the HRS.

For example, the publication by Hors-Fraile et al.^31^ identified that although their HRS was not grounded in BCT, it has potential to “improve effectiveness to adopt and maintain healthy habits” by integrating BCT. The work by Zavyalova et al.^25^ was motivated by changing and “correcting” hypertension treatment adherence as a form of behavior change but shares no discussion pertaining to BCT. Similarly, Afonso et al.^34^ recognized from their study results that their current HRS design on its own is insufficient to motivate behavior change. Only one publication from Torkamaan and Ziegler^41^ more broadly discusses implementation guidelines for HRSs to specifically integrate BCT and persuasive design principles for health promotion. These guidelines are presented as a set of requirements that developers and designers can follow when creating HRSs.

### Connections between shared decision-making and HRS purpose should be more explicit

Across publications, there is consensus that the purpose of an HRS, no matter the health domain, is to help the patient become more involved in managing their health. Multiple (8/45, 19.0%) publications have referred to shared decision-making, but to varying extents. Five of the eight articles (5/8, 62.5%) provide definitions for shared decision-making and offer brief connections describing how their HRS fulfills that definition, while the other three (3/8, 37.5%) articles more explicitly situate the tool in a shared decision-making context.

HRSs that provide educational knowledge and recommendations, such as the ones proposed by Iatraki et al.^23^ and Jacobs et al.^,29^ define the end goal of shared decision-making as the patient being better informed and as a result, more involved in the decision-making process. Luna-Aveiga et al.^24^ distinguish between self-management and patient self-care, explaining that the emphasis of the latter is about the patient only, whereas the former encourages interaction between patients and physicians. Medina-Moreira et al.^18^ share a similar framing, as they describe shared decision-making being based on the extent a healthcare professional can use patient data to become better informed about the patient. In a more general sense, Mustaqeem et al.^21^ broadly speak to the idea of shared decision-making by explaining that an HRS can help both the patients and physicians identify recommendations in the context of cardiac disease.

In contrast, three other publications elaborate on the use of their HRS in a shared decision context. Wang et al.^19^ motivate their work by situating their HRS in the “three talk model of shared decision making” framework,^58^ explaining that the HRS can help prepare patients to participate in making choices regarding their treatment plans during consultations with their physician. Ran et al.^49^ choose to focus their future work on identifying whether their HRS can be an effective way to unify the views of both patients and their care teams to enable shared decision-making. Lastly, Gräßer et al.^20^ discuss the use of an HRS to facilitate integration of patient values and preferences during the shared decision-making process. Future work should continue to integrate the concept of shared decision-making early on in the design stage and throughout the development process.

### HRSs can often offer other self-management services, such as progress tracking and chatbots

Besides providing a recommendation, an HRS also has the potential to integrate with other patient-facing, self-management services. For example, the ability for users to track their progress by accessing their data history was explicitly described in the work presented by Chen, Godinho, Hales, and Rohani.^15,37,39,56^ Providing an extra feature such as self-tracking is not only meant to inform the patient, as outlined by Chen et al.,^56^ but also to motivate the user to be more active in managing their health. Other services, such as a chatbot, provide additional features to the user beyond a recommendation; for example, the chatbot described by Ishraque et al.^26^ relays the results from the HRS through conversation. This work also has another feature which provides the user with visualizations as a way for the user to better understand their current health condition and goals. The HRS by Hales et al.^15^ not only enables patients seeking weight loss to track their diet, physical activity, weight, and goals, it also presents a news feed that shares the patients’ progress with others, allowing users to send notes of positive encouragement when those goals are met. The design decisions related to the system presented by Hales et al. are specifically embedded in BCT. Lastly, the work shared by Chaturvedi et al.^52^ described an HRS as just one component of a larger mental health platform that provided other features such as modules for user self-care, a content library, and a chatbot (both text and video options). Results from their study indicate that the content included in the HRS section specifically had higher completion rates by users than the other sections listed above, which the user was free to access on their own time. While this may suggest that users derive more benefit from an HRS service compared to other features, these findings are likely situated in their target domain, population, and motivation.

## Discussion

### Main findings

We identified 45 publications pertaining to the design, development, and/or evaluation of HRSs for chronic disease management since 2010. This review addresses the existing gap of knowledge in the literature regarding HRS use for chronic disease management, by shifting the focus from specific, technology-focused HRS attributes to identifying the progress made on their use in a broader systems level (study aims 1 and 2) and also reports on the extent of use and implementation in real-life settings (study aim 3).

#### What are the key design characteristics used in an HRS that help create user personalization?

The first aim of this scoping review was to identify the key design characteristics of the HRS as a way to determine level of personalization. Given the primary purpose of an HRS, the recommended output to the user should be personalized enough so that it is relevant and helpful. If the user finds the suggestions helpful, there is a higher likelihood of them continually using the tool when needed to help guide their decision-making. While some HRSs had elaborate data inputs, most collected only the essentials: demographic, and basic health profile. This observation may be attributed to a lack of access to the comprehensive data needed for personalized recommendations. The challenge of interoperability in healthcare may constrain HRSs from being as sophisticated as they could be.

#### What are the main tools and processes used during HRS design and development?

The second aim of the scoping review—to identify the use of tools and processes in the design and development process—could be used to inform the type of data that should be collected so that the recommendations are beneficial to the user. To help HRSs enact behavior change, BCT can be (and in some cases has been) integrated to offer more personalized suggestions and to increase engagement with the HRS itself. Besides Torkamaan and Ziegler,^41^ who shared a framework to integrate BCT into HRSs, the actual use of these theories is fragmented despite there being consensus on the importance of user engagement with and effectiveness of the HRS. In the chronic self-management health domain, continual use of a tool such as an HRS is required in order for patients to successfully self-manage their disease. Taken together, a lack of BCTs and data inputs limits the progress of RS use in the health domain space. Similarly, evaluations should begin to focus on understanding HRS effectiveness by investigating the influence they have on user behavior.

#### What is the intended use of HRSs and extent of their clinical integration?

The third aim of the scoping review investigated the intended use of the HRS and the extent of current integration into clinical practice. The findings from this review identified a discrepancy between the described intended use of each HRS and the focus of the published research findings. The focus of HRS research should shift from algorithmic evaluations to studies that better understand their effectiveness (thematic analysis finding #1). Although an HRS is meant to bring about a specific behavior change, most publications presented formative work and were not yet at the stage to explore this research area further. Publications that had the most amount of evidence from late-stage evaluations like RCTs mostly provided motivational messaging to the user and were still limited in analyzing behavioral change from the user; instead, these publications focused on ratings of relevance and other self-reported measures from users. While formative work and algorithmic evaluations are necessary development steps before deploying an HRS into real-world settings, researchers must also be willing to take this next step in deploying their systems so that we may learn more about HRS effectiveness. This sentiment inherently requires better integration of theories, models, frameworks, and/or guidelines in the development process, to systematically work towards better and more effective HRSs (thematic analysis finding #2).

The use and implementation of these systems into clinical practice also requires intentional planning and consideration, extending beyond just setting up access for the user. As mentioned in the thematic analysis, some publications in this review referred to shared decision-making when describing a specific HRS (thematic analysis finding #3). Depending on the HRS output type (e.g. educational information and pharmaceutical recommendations), there is potential for HRSs to further inform patients regarding their health management. For example, this could include prompting patients to learn more about a specific topic or to identify important aspects to further discuss with their care team. Patients who are better informed can have better shared decision-making experiences, so in addition to the HRS eliciting specific behavior change from the user, the platform can also set the patient up to engage with their care providers in a way that they may not have been able to prior to interacting with the tool. HRSs can do more than provide personalized and accurate suggestions; they can also serve as a powerful way of encouraging patient involvement in their healthcare discussions (thematic analysis finding #4). Future work should therefore consider how a platform such as the HRS can be implemented within current workflow practices so that patients make good use of the output and their experience when managing their health with their broader clinical team.

#### Looking ahead: deterministic versus nondeterministic recommender systems

The findings shared in this review, regarding the importance and lack of processes to design and evaluate HRS systems, are especially relevant given the recent, rapid developments in generative artificial intelligence (AI). Shifting the underlying RS infrastructure from deterministic to nondeterministic (i.e. generative AI) works towards outputs with higher accuracy and better personalization for the user, thus leading to greater effectiveness of the tool. This shift in technology is impactful for content-based RSs, which rely on mapping item contents to user preferences. Having a pre-trained model with a vast knowledge base (e.g. large language models) provides an opportunity for the RS to perform the same, if not better, with mapping item characteristics to user preferences.^59^ As the field of generative AI continues to improve and become more accessible, it is expected that RS performance will also be enhanced, thus creating a tool that is more useful to the end user. Major AI concerns, such as transparency and explainability, contribute to the growing need and urgency of developing standardized processes for the development of HRSs.

## Limitations

This review had several limitations, each of which can be related to either the process that was followed or to the contents included in the scoping review.

### Limitations related to publications

During the review process, some publications were initially considered relevant but were eventually excluded because they did not share sufficient details surrounding the HRS design. As a result, this review may not have included all applicable HRSs for chronic disease. Another limitation is that some publications were excluded because the HRS was for the wrong user population (clinicians) but were otherwise relevant. Lastly, all the data shared in this review were derived exclusively from the publications, so there may still be study details that are relevant to this publication but inaccessible due to the authors’ decisions of what to include.

### Limitations related to process

First, limiting this review to only chronic diseases meant that we were unable to draw conclusions about recommender systems in the general health domain, rather, we are only able to comment on the work done so far with select chronic diseases. Second, despite both reviewers independently creating and testing search strategies prior to reaching consensus on the best search strategy to move forward with, some publications relevant to this domain may have been missed. Third, constraining the language of peer-reviewed publications to English may have caused us to not include relevant work that is shared in another language. Also, limiting the review to peer-reviewed publications could have omitted research published elsewhere, which is especially relevant given the pace of developments in this field.

## Conclusions

This review works towards situating HRSs as a tool for collaborative decision-making in the chronic disease space, by presenting details pertaining to their use through a broader systems-level lens than previous scoping reviews (by identifying key design characteristics, main processes and theories followed, as well as current use and implementation). The publications in this review showcase that the potential HRSs have to offer personalized health recommendations to patients, thereby improving their participation and collaboration in disease management (behavior change), but research needs to further investigate their effectiveness in doing so. The goal of creating HRSs that are effective tools for collaborative decision-making can be supported by better implementing relevant theories, models, and frameworks during the design and development process and conducting studies that focus on ensuring they fulfill their intended use.

## Supplemental Material

sj-docx-1-dhj-10.1177_20552076241309386 - Supplemental material for Health recommender systems to facilitate collaborative decision-making in chronic disease management: A scoping review Supplemental material, sj-docx-1-dhj-10.1177_20552076241309386 for Health recommender systems to facilitate collaborative decision-making in chronic disease management: A scoping review by Antonia Barbaric, Kenneth Christofferson, Susanne M Benseler, Chitra Lalloo, Alex Mariakakis, Quynh Pham, Joost F Swart, Rae S M Yeung and Joseph A Cafazzo in DIGITAL HEALTH

sj-docx-2-dhj-10.1177_20552076241309386 - Supplemental material for Health recommender systems to facilitate collaborative decision-making in chronic disease management: A scoping review Supplemental material, sj-docx-2-dhj-10.1177_20552076241309386 for Health recommender systems to facilitate collaborative decision-making in chronic disease management: A scoping review by Antonia Barbaric, Kenneth Christofferson, Susanne M Benseler, Chitra Lalloo, Alex Mariakakis, Quynh Pham, Joost F Swart, Rae S M Yeung and Joseph A Cafazzo in DIGITAL HEALTH

sj-docx-3-dhj-10.1177_20552076241309386 - Supplemental material for Health recommender systems to facilitate collaborative decision-making in chronic disease management: A scoping review Supplemental material, sj-docx-3-dhj-10.1177_20552076241309386 for Health recommender systems to facilitate collaborative decision-making in chronic disease management: A scoping review by Antonia Barbaric, Kenneth Christofferson, Susanne M Benseler, Chitra Lalloo, Alex Mariakakis, Quynh Pham, Joost F Swart, Rae S M Yeung and Joseph A Cafazzo in DIGITAL HEALTH
