# Supplementary material for: Health recommender systems to facilitate collaborative decision-making in chronic disease management: A scoping review
Source: Digit Health. 2025 Jan 6;11:20552076241309386. doi: 10.1177/20552076241309386 (PMC11705346; doi:10.1177/20552076241309386)
Supplement: sj-docx-2-dhj-10.1177_20552076241309386 - Supplemental material for Health recommender systems to facilitate collaborative decision-making in chronic disease management: A scoping review [file sj-docx-2-dhj-10.1177_20552076241309386.docx]

Appendix S2: Example search string in MEDLINE

1. (“recommender system*” or “recommendation system*” or “recommender engine*” or “recommendation engine*” or (recommender system* adj3 health*)).mp. [mp=title, book title, abstract, original title, name of substance word, subject heading word, floating sub-heading word, keyword heading word, organism supplementary concept word, protocol supplementary concept word, rare disease supplementary concept word, unique identifier, synonyms]
2. exp. Decision Support Systems, Clinical/ or exp Decision Support Techniques/ or exp Decision Making, Computer-Assisted/
3. (“choice” or “decision*” or “decision making” or “decision support” or “clinical decision support” or “support tool” or “prefer*” or “need” or “person*” or “usage” or “attitud*” or “assist*” or “guid*” or “self-management” or “health promotion” or “behaviour” or “behavior” or “readiness*”).mp. [mp=title, book title, abstract, original title, name of substance word, subject heading word, floating sub- heading word, keyword heading word, organism supplementary concept word, protocol supplementary concept word, rare disease supplementary concept word, unique identifier, synonyms]
4. exp Therapeutics/
5. exp Medication Therapy Management/
6. exp Chronic Disease/
7. exp Mental Health/
8. exp Smoking/ or exp Alcoholism/ or exp Behavior, Addictive/ or exp Substance- Related Disorders/
9. exp Health Promotion/

10. (health* or clinic* or therap* or “treatment plan” or “drug*” or “service*” or “medicat*” or “prescrip*”).mp. [mp=title, book title, abstract, original title, name of substance word, subject heading word, floating sub-heading word, keyword heading word, organism supplementary concept word, protocol supplementary concept word, rare disease supplementary concept word, unique identifier, synonyms]

11. 4 or 5 or 6 or 7 or 8 or 10

12. 2 or 3 or 9

13. 1 and 11 and 12
