# Supplementary material for: Health recommender systems to facilitate collaborative decision-making in chronic disease management: A scoping review
Source: Digit Health. 2025 Jan 6;11:20552076241309386. doi: 10.1177/20552076241309386 (PMC11705346; doi:10.1177/20552076241309386)
Supplement: sj-docx-3-dhj-10.1177_20552076241309386 - Supplemental material for Health recommender systems to facilitate collaborative decision-making in chronic disease management: A scoping review [file sj-docx-3-dhj-10.1177_20552076241309386.docx]

| **Title** | **Lead author** | **Year of**  **publication** | **Location of**  **publication** | **Research**  **Methodology** | **Type of User**  **Involvement** | **Health Domain** | **Intended User** | **HRS Output Type** | **Data Inputs** | **Source of Data Inputs** | **Theories Guiding HRS Design** |
| --- | --- | --- | --- | --- | --- | --- | --- | --- | --- | --- | --- |
| Design and Field Evaluation of REMPAD: A Recommender System Supporting Group Reminiscence Therapy | Yang et al. | 2013 | Ireland | UCD | Active: participates in a clinical trial | Dementia | Patient with clinician | Education | User Demographic; Lifestyle; Feedback about recommendation | User | User centered design; |
| Towards collaborative filtering recommender systems for tailored  health communications. | Marlin et al. | 2013 | United States | Design | Active: provides feedback | Tobacco Dependence | Patient with clinician | Motivational msging | Feedback about recommendation | User | None |
| A Mobile-Based Tailored Recommendation System for Parents of Children with Overweight or Obesity: A New Tool for Health Care Centers. | Afonso et al. | 2020 | Portugal | Pilot study | Active: participates in a clinical trial | Obesity | Patient with caregiver | Education | Lifestyle | User | Behavior Change Theories; |
| Evaluation of a recommender app for apps for the treatment of depression and anxiety: an analysis of longitudinal user engagement. | Cheung et al. | 2018 | United States | Observational | Active: participates in a clinical trial | Mental health | Patient | Digital intervention | Unknown | Unknown | None |
| Comparison of a Collective Intelligence Tailored Messaging System on Smoking Cessation Between African American and White People Who Smoke: Quasi- Experimental Design. | Faro et al. | 2020 | United States | Pilot Study | Active: participates in a clinical trial | Tobacco Dependence | Patient | Motivational msging | Feedback about recommendation | User; Dataset of past user activity | Behavior Change Theories; Guidelines from authoritative sources |
| Effect of a Machine Learning Recommender System and Viral Peer Marketing Intervention on Smoking Cessation: A Randomized Clinical Trial. | Faro et al. | 2023 | United States | RCT | Active: participates in a clinical trial | Tobacco Dependence | Patient | Motivational msging | Feedback about recommendation | Datasets of past user activity | Behavior Change Theories; Guidelines from authoritative sources |
| Fynex: Work in Progress on a Web-based Approach That Implements a Hybrid Recommendation System for Preventing and Treating Diseases based on Eating Disorders | Gonzalez and Feijóo- García | 2022 | Colombia | Pilot Study | Active: participates in a clinical trial | Eating disorder | Patient with clinician | Lifestyle: diet | User Demographic; Health Profile | User | None |
| Serious games for rehabilitation: Gestural interaction in personalized gamified exercises through a recommender system. | Gonzalez-Gonzalez et al. | 2019 | Spain | Pilot Study | Active: participates in a clinical trial | Physical/Motor Disability: Rehab | Patient with clinician | Lifestyle: rehab | User Demographic, Feedback about recommendation; Lifestyle | User; Medical Device | Gamification Theory; |
| Social networks for improving healthy weight loss behaviors for overweight and obese adults: A randomized clinical trial of the social pounds off digitally (Social POD) mobile app. | Hales et al. | 2016 | United States | RCT | Active: participates in a clinical trial | Obesity | Patient | Motivational msging | Health Profile; Lifestyle | User | Behavior Change Theories; |
| Usability Evaluation of an Adaptive Information Recommendation System  for Breast Cancer Patients. | Jacobs et al. | 2019 | United States | Usability study | Active: participates in a clinical trial | Cancer | Patient | Education | Health Profile | User | None |
| Benefits of Using Activity Recommender Technology for Self-management of  Depressive Symptoms | Rohani et al. | 2021 | Denmark | Feasibility | Active: participates in a clinical trial | Mental health | Patient | Intervention specific | User Demographic; Mental Health | User; | Behavior Change Theories; |
| Impact of a Collective Intelligence Tailored Messaging System on Smoking Cessation: The Perspect Randomized Experiment. | Sadasivam et al. | 2016 | United States | RCT | Active: participates in a clinical trial; Active: provides feedback | Tobacco Dependence | Patient | Motivational msging | User Demographic; Lifestyle; Feedback about recommendation | User; dataset of past user activity | Behavior Change Theories; Guidelines from authoritative sources |
| Evaluating the use of a recommender system for selecting optimal messages for smoking cessation: patterns and effects of user-system engagement. | Chen et al. | 2021 | United States | Observational | Active: provides feedback | Tobacco Dependence | Patient | Motivational msging | Feedback about recommendation; | User; dataset of past user activity | Guidelines from authoritative sources; |
| Dissemination and Effectiveness of the Peer Marketing and Messaging of a Web- Assisted Tobacco Intervention: Protocol for a Hybrid Effectiveness Trial. | Faro et al. | 2019 | United States | Design | No User Involvement | Tobacco Dependence | Patient | Motivational msging | Feedback about recommendation; | User; dataset of past user activity | Behavior Change Theories; Guidelines from authoritative sources |
| Designing a personalised case-based recommender system for mobile self- management of diabetes during exercise | Chen et al. | 2017 | Scotland | Design | Active: participates in a clinical trial | Diabetes | Patient | Lifestyle: diet, physical activity | User Demographic; Health Profile; Lifestyle | User; dataset of past user activity | None |
| A smartphone-based personalized activity recommender system for  patients with depression | Hung et al. | 2015 | Taiwan | Pilot Study | Active: participates in a clinical trial | Mental health | Patient | Intervention specific | Feedback about recommendation; User Demographic; Lifestyle | User; Medical Device | None |
| Astmapp: A platform for asthma self-  management | Luna-Aveiga et al. | 2018 | Ecuador | Usability study | Active: participates in  a clinical trial | Asthma | Patient with clinician | Education | Health Profile | User; dataset of past user  activity | None |
| A collaborative filtering based recommender system for disease self-  management | Medina-Moreira et al. | 2017 | Ecuador | Design | Active: provides feedback | Diabetes | Patient with clinician | Lifestyle: diet | User Demographic; Health Profile; Lifestyle | User; Dataset of past user activity | Guidelines from authoritative sources; |

| Explainable Artificial Intelligence Recommendation System by Leveraging the Semantics of Adverse Childhood Experiences: Proof-of-Concept Prototype Development. | Ammar and Shaban- Nejad | 2020 | United States | Design | No User Involvement | Mental health | Patient, Caregiver, Clinician | Intervention specific | Mental Health Profile | User | Guidelines from authoritative sources; |
| --- | --- | --- | --- | --- | --- | --- | --- | --- | --- | --- | --- |
| A Recommendation System of Nutrition and Physical Activity for Patients with  Type 2 Diabetes Mellitus | Godinho et al. | 2020 | Portugal | Design | No User Involvement | Diabetes | Patient | Lifestyle: diet | User Demographic; Lifestyle; Feedback about recommendation | User | None |
| Drugs Rating Generation and Recommendation from Sentiment Analysis of Drug Reviews using Machine Learning | Hossain et al. | 2020 | New Zealand | Design | No User Involvement | General Purpose | Patient | Drug intervention | External source | Datasets of past user activity | None |
| HELPeR: An Interactive Recommender System for Ovarian Cancer Patients  andCaregivers | Rahdari et al. | 2022 | United States | Design | No User Involvement | Cancer | Patient with caregiver | Education | Health Profile | User; Dataset of past user activity | None |
| Integrating Behavior Change and Persuasive Design Theories into an ExampleMobile Health Recommender System | Torkamaan and Ziegler | 2021 | Germany | Design | No User Involvement | Mental health | Patient | Intervention specific | Health Profile; Lifestyle | Unknown | Behavior Change Theories; |
| Personal Health Information Recommender: implementing a tool for the empowerment of cancer patients. | Iatraki et al. | 2018 | Greece | Pilot Study | Active: participates in a clinical trial | Cancer | Patient | Education | Health Profile; Lifestyle; Feedback about recommendation | Unknown | None |
| Basic principles for the development of an AI-based tool for assistive technology  decision making. | Ran et al. | 2022 | United States | Recommendation paper | No User Involvement | Physical/Motor Disability: Rehab | Patient with clinician | Lifestyle: assistive tech | Unknown | Unknown | None |
| MED-StyleR: METABO Diabetes-Lifestyle  Recommender | Hammer et al. | 2010 | Spain | Design | No User Involvement | Diabetes | Patient | Lifestyle: diet, physical  activity | Lifestyle; Health Profile | Unknown | None |
| Designing a mobile recommender system for treatment adherence  improvement among hypertensives | Zavyalova et al. | 2018 | Russia | Design | No User Involvement | Cardiac | Patient | Motivational msging | Lifestyle; Health Profile; Mental Health | User; Medical Device | Behavior Change Theories; |
| Individualized patient-centered type 2  diabetes recommender system | Afreen et al. | 2019 | India | Design | Passive: used for data  purposes only | Diabetes | Patient | Lifestyle: diet | User Demographic; Health Profile | User; | None |
| Facilitating CPAP adherence with personalized recommendations using  artificial neural networks | Araujo et al. | 2021 | United States | Observational | Passive: used for data purposes only | Obstructive sleep apnea | Patient | Drug intervention | Health Profile | Medical Device; | None |
| Content Recommendation Systems in Web-Based Mental Health Care: Real- world Application and Formative Evaluation. | Chaturvedi et al. | 2023 | United States | Observational | Passive: used for data purposes only | Mental health | Patient | Education | Mental Health Profile | User; | None |
| Improving Outcomes Through Personalized Recommendations in a Remote Diabetes Monitoring Program: Observational Study. | Kamath et al. | 2022 | United States | Observational | Passive: used for data purposes only | Diabetes | Patient | Intervention specific | User Demographic; Health Profile; Lifestyle | User; | None |
| Can a Recommender System Support Treatment Personalisation in Digital Mental Health Therapy? A Quantitative Feasibility Assessment Using Data from a Behavioural Activation Therapy App | Lewis et al. | 2022 | United States | Proof of Concept Experiments | Passive: used for data purposes only | Mental health | Patient | Intervention specific | Feedback about recommendation; | User; Dataset of past user activity | None |
| A Shared Decision-Making System for Diabetes Medication Choice Utilizing Electronic Health Record Data | Wang et al. | 2017 | China | Design | Passive: used for data purposes only | Diabetes | Patient with clinician | Drug intervention | User Demographic; Health Profile | Electronic health records | International Patient Decision Aids Standard; Three-step shared decision making model |
| Opening the Black Box: Explaining the Process of Basing a Health Recommender System on the I-Change Behavioral Change Model | Hors-Fraile et al. | 2019 | Taiwan | Design | Passive: used for data purposes only; Active: participates in a clinical trial | Tobacco Dependence | Patient | Motivational msging | User Demographic; Health Profile | Unknown | Behavior Change Theories; |
| Therapy Decision Support Based on  Recommender System Methods. | Graber et al. | 2017 | Germany | Design | Passive: used for data  purposes only | Psorasis | Patient with clinician | Drug intervention | User Demographic; Health Profile | Electronic health records | None |
| A statistical analysis based recommender model for heart disease  patients. | Mustaqeem et al. | 2017 | Pakistan | Feasibility | Passive: used for data purposes only | Cardiac | Patient with clinician | Intervention specific | User Demographic; Health Profile | Electronic health records | None |
| A Novel Rule-Based Recommender System For The Indian Elderly Diabetic Population | Ramesh et al. | 2021 | India | Design | Passive: used for data purposes only; Active: participates in a clinical trial | Diabetes | Patient | Lifestyle: diet | Health Profile | User; | None |
| emHealth: Towards Emotion Health Through Depression Prediction and Intelligent Health Recommender System | Yang et al. | 2018 | China | Case vignettes | Passive: used for data purposes only; Simulated data | Mental health | Patient | Intervention specific | Mental Health Profile; Lifestyle | User; | Guidelines from authoritative sources; |

| A fuzzy recommendation system for the automatic personalization of physical rehabilitation exercises in stroke patients | Gmez-Portes et al. | 2021 | United Kingdom | Design | Simulated data | Physical/Motor Disability: Rehab | Patient with clinician | Lifestyle: rehab | Health Profile | User; Medical Device | None |
| --- | --- | --- | --- | --- | --- | --- | --- | --- | --- | --- | --- |
| Artificial intelligence-based cardiac rehabilitation therapy exercise  recommendation system | Ishraque et al. | 2018 | Canada | Design | Simulated data | Physical/Motor Disability: Rehab | Patient with caregiver | Lifestyle: rehab | User Demographic; Health Profile; Lifestyle | User; | None |
| Personalized Adaptive CBR Bolus Recommender System for Type 1  Diabetes. | Torrent-Fontbona and Lopez | 2019 | Spain | Design | Simulated data | Diabetes | Patient | Drug intervention | Lifestyle; Health Profile | Medical Device; | None |
| A pharmaceutical therapy recommender system enabling shared  decision-making. | Graber et al. | 2022 | Germany | Design | No User Involvement | Psorasis | Patient with clinician | Drug intervention | User Demographic; Health Profile | Electronic health records | Guidelines from authoritative sources; |
| Usability Evaluation of a Knowledge Graph-Based Dementia Care Intelligent Recommender System: Mixed Methods Study | Leng et al. | 2023 | China | Usability Study | Passive: used for data purposes only; Active: participates in a clinical trial | Dementia | Caregiver with clinician | Intervention specific | User Demographic; Health Profile; Lifestyle | User | Guidelines from authoritative sources; |
| The Implementation of Recommender Systems for Mental Health Recovery Narratives: Evaluation of Use and Performance | Slade et al. | 2024 | United Kingdom | Feasibility | Active: provides feedback | Mental health | Patient | Intervention specific | Feedback about recommendation | User | None |
| Evaluating the Effectiveness of a Generative Pretrained Transformer- Based Dietary Recommendation System in Managing Potassium Intake for Hemodialysis Patients | Jin et al. | 2024 | China | Proof of Concept Experiments | Active: participates in a clinical trial | Kidney/End Stage Renal Disease | Patient | Lifestyle: diet | Lifestyle | User; External Knowledge Base | None |
